# Seroepidemiology of Human Enterovirus 71 Infection among Children, Cambodia

**DOI:** 10.3201/eid2201.151323

**Published:** 2016-01

**Authors:** Paul F. Horwood, Alessio Andronico, Arnaud Tarantola, Henrik Salje, Veasna Duong, Channa Mey, Sovann Ly, Philippe Dussart, Simon Cauchemez, Philippe Buchy

**Affiliations:** Institut Pasteur in Cambodia, Phnom Penh, Cambodia (P.F. Horwood, A. Tarantola, V. Duong, C. Mey, P. Dussart, P. Buchy);; Institut Pasteur, Paris, France (A. Andronico, H. Salje, S. Cauchemez);; Johns Hopkins Bloomberg School of Public Health, Baltimore, Maryland, USA (H. Salje);; Ministry of Health, Phnom Penh (S. Ly);; GlaxoSmithKline Vaccines, Singapore (P. Buchy)

**Keywords:** enterovirus, EV71, encephalitis, seroprevalence, Cambodia, viruses, children

## Abstract

Enterovirus 71 is reported to have emerged in Cambodia in 2012; at least 54 children with severe encephalitis died during that outbreak. We used serum samples collected during 2000–2011 to show that the virus had been widespread in the country for at least a decade before the 2012 outbreak.

In the Asia-Pacific region, human enterovirus 71 (EV71) is a widespread pathogen that causes hand, foot and mouth disease among children. Potentially fatal neurologic and systemic manifestations develop in a small proportion of patients (*1*).

In Cambodia during 2012, a disease outbreak characterized by severe encephalitis with cardiovascular collapse and pulmonary edema seized international headlines and resulted in the death of at least 54 children; EV71 subgenogroup C4 was identified as the cause (*2*). The large number of deaths during a short period was a concern for health authorities. To investigate whether EV71 had circulated in Cambodia before the 2012 outbreak, we retrospectively screened blood samples collected from children during 2000–2011. 

## The Study

We screened serum samples collected from inpatient children in Cambodia through routine national dengue surveillance. The study set was extracted from the Institut Pasteur in Cambodia biobank of strictly anonymized samples collected from 9,408 febrile inpatients during 2000–2011. Ethics clearance was obtained from the Cambodian National Ethics Committee for Human Research before testing commenced.

After exclusion of data entry errors, outliers in terms of year of participation, and insufficient data or samples, the database included 7,823 children 2–15 years of age for whom age, sex, and province of residence were documented. To avoid any influence from maternal antibodies, we excluded children <2 years of age from the study. Provinces were allocated to geographic quadrants and mapped by using ArcGIS 10 (Esri Co., Redlands, CA, USA) (Technical Appendix Figure 1). Random sampling was applied by using Stata 11 (StataCorp LP, College Station, TX, USA) with a representation of samples for each year. A total of 1,707 anonymized samples (1 sample/child) were selected and tested. All available samples from the sparsely populated northeastern quadrant (4% of the dataset) were included. Because the southeastern quadrant (bordering Vietnam) is the most populated quadrant, samples were selected in approximate proportion to population (46% of the dataset). Samples from the southwestern (18% of dataset) and northwestern (31% of the dataset) quadrants, each of which borders Thailand, were selected proportionally to represent a total of 48% from the quadrants bordering Thailand (Table).

**Table:** Population statistics and enterovirus 71 seroprevalence among children 2–15 years of age, by geographic quadrant, Cambodia, 2000–2011

Quadrant	Population (% seroprevalence)*	No. participants (% of dataset)	Overall seroprevalence, no. (%)
Northwest	2,785,308 (20.2)	535 (31.3)	488 (91.2)
Northeast	1,445,009 (10.5)	76 (4.4)	64 (84.2)
Southwest	3,831,832 (27.8)	314 (18.4)	271 (86.3)
Southeast	5,703,047 (41.4)	782 (45.8)	693 (88.6)
Total	13,765,196 (100)	1,707 (100)	1,516 (88.8)

*All-age population in 2008 (census nearest to retrospective study midpoint). Data from the National Institute of Statistics, Directorate General for Health, Cambodia Demographic ICF Macro, 2011; General Population Census of Cambodia, 2008; and Ministry of Planning, 2009.

The 1,707 serum samples were screened by use of a microneutralization assay to detect neutralizing antibodies against an EV71 strain (genotype C4a) isolated from an infected child during the 2012 outbreak in Cambodia. The assay was conducted on Vero E6 cells by mixing 2-fold serial dilutions (1:8 to 1:8,192) of heat-inactivated human serum samples with 100 μL (2,000 50% tissue culture infective doses/mL) of the EV71 strain. Cytopathic effect was determined visually before and after staining with 2.5% crystal violet solution. All serum samples were tested in duplicate, and positive control serum was added to each reaction plate for quality control purposes. The lowest dilution at which cytopathic effect was observed in >50% of wells was considered the antibody titer of the serum sample. A titer of >1:16 was considered the cutoff for a positive antibody response and was a more stringent cutoff than that used in previously published EV71 seroprevalence studies, in which the cutoff was usually >1:8 (*3*–*6*).

To reconstruct the historical annual probability of infection, we used information about the serostatus and age of the children. This reconstruction assumed that after infection, detectable antibody titers are long lasting; this method has been used to estimate the historical force of infection for other diseases, such as dengue (*7*). We estimated a separate annual probability of infection for each year from 1994 through 2011. Because no patients in our dataset had been born before 1994, we could not estimate the force of infection before this time (online Technical Appendix). Because samples included in this study were from children with a denguelike illness, rates of EV71 infection among study participants might not accurately reflect rates among all children in Cambodia. However, because an average of 87.8% of patients recruited by the National Dengue Surveillance Program had a laboratory-confirmed dengue infection (*8*), the febrile episode that triggered the hospitalization could be only slightly associated with EV71 infection and thus would have negligible influence on the EV71 neutralizing titers of the patient population.

**Figure 1:**
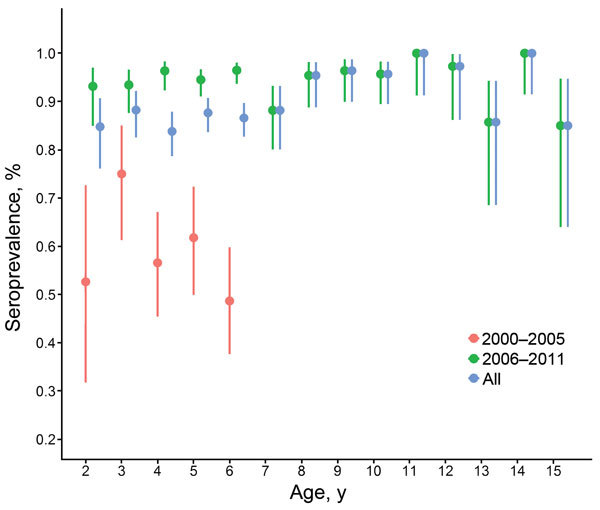
Age-associated seroprevalence of enterovirus 71 (EV71) infection in Cambodia, estimated by detection of EV71 seroneutralizing antibodies in inpatient children 2–15 years of age, 2000–2011. Error bars indicate 95% CIs. Serum samples were collected from routine national dengue surveillance in Cambodia.

Among children in this study, the overall seroprevalence of EV71 neutralizing antibodies was 88.8%: 1,300 (94.8%) of 1,371 (95% CI 93.5%–95.9%) among children 2–15 years of age sampled during 2006–2011 and 216 (64.3%) of 336 (95% CI 58.9%–69.4%) among those 2–7 years of age sampled during 2000–2005 (Figure 1). Seroprevalence did not vary substantially by age group. This profile across age groups remained unchanged in more stringent analyses with higher cutoff values (Technical Appendix Figure 2) in which, despite levels of seropositivity decreasing with higher cutoff titers, the reduction was consistent across all age groups. Seroprevalence of EV71 relative to sex did not differ significantly (89.8% among girls vs. 87.7% among boys; p = 0.18).

**Figure 2:**
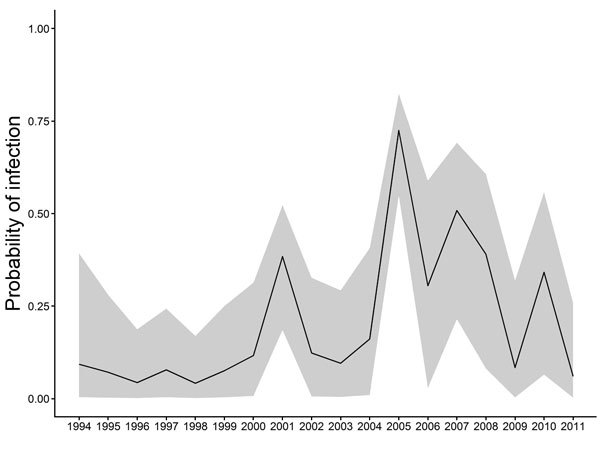
Annual probability of enterovirus 71 infection (EV71) in Cambodia during 1994–2011, estimated by detection of EV71 seroneutralizing antibodies in inpatient children 2–15 years of age. Serum samples were collected from routine national dengue surveillance in Cambodia.

Epidemic curves derived from the seroprevalence data show the dynamics of infection for the whole country (Figure 2) and across the 4 quadrants (Technical Appendix Figure 3). The reconstructed curves were coherent, showing large-scale, countrywide circulation of the virus since 2002. Seroprevalence peaks every 2–3 years indicate a cyclical pattern of EV71 outbreaks. This pattern has been reported from other Asia-Pacific countries (*9*–*11*) and probably represents the time needed for establishment of a new cohort of immunologically naive patients. In countries with a larger population, such as China, infection might peak annually (*12*).

Despite our use of a more stringent cutoff value, the seroprevalence detected in our study was considerably higher than that reported from previous studies in the region, during which a cutoff of 1:8 was invariably used (*3*–*6*). If we had used a neutralization titer of 1:8, seroprevalence would have been 93.1% (n = 1,590 positive samples). Intense circulation of EV71 was therefore occurring in Cambodia long before the 2012 outbreak.

In Cambodia and other Asia-Pacific countries, other enteroviruses commonly cocirculate with EV71. Some of these strains, such as coxsackieviruses A6 and A16, have also been associated with severe neurologic illnesses in children. Previous studies have established that cross-neutralization occurs among different EV71 strains and genogroups (*13*,*14*). However, there is no evidence of cross-neutralization between EV71 and other enteroviruses (*15*). Cross-neutralization at high dilutions would probably not have generated a consistent profile of seropositivity across children of different ages (Technical Appendix Figure 2). Thus, the high level of seropositivity observed in this study is probably specific for EV71.

## Conclusions

Our data support the widespread circulation of EV71 at least a decade before its reported emergence in 2012. Furthermore, reconstructed epidemic curves suggest that EV71 outbreaks occurred in a cyclical pattern in Cambodia and that the virus infected large proportions of immunologically naive children every 2–3 years. Before 2012, this circulation remained undetected, highlighting the need to further reinforce the surveillance systems in developing countries. Also needed is enhanced medical education for better identification of infectious diseases such as hand, foot and mouth disease, which, despite its association with relatively specific clinical signs, requires careful physical examination of patients. 

It is still unknown why so many severe cases were detected during the 2012 EV71 outbreak in Cambodia. However, seroepidemiologic studies in other settings have also confirmed widespread circulation before outbreaks (*5*,*9*). For combatting this pathogen, developments in vaccines and antiviral drugs are urgently needed.

**Technical Appendix.** Enterovirus 71 among children 2–15 years of age, Cambodia. Distribution of provinces into quadrants, age-associated seroprevalence, estimated annual probability of infection by geographic quadrant during 1994–2011, and statistical model used to estimate annual probability.
