# Supplementary material for: Seroepidemiology of Human Enterovirus 71 Infection among Children, Cambodia
Source: Emerg Infect Dis. 2016 Jan;22(1):92–5. doi: 10.3201/eid2201.151323 (PMC4696711; doi:10.3201/eid2201.151323)
Supplement: Supplementary file 1 — Technical Appendix. Enterovirus 71 among children 2–15 years of age, Cambodia. Distribution of provinces into quadrants, age-associated seroprevalence, estimated annual probability of infection by geographic quadrant during 1994–2011, and statistical model used to estimate annual probability. [file 15-1323-Techapp-s1.pdf]

Article DOI: http://dx.doi.org/10.3201/eid2201.151323

Seroepidemiology of Human Enterovirus 71 Infection among Children, Cambodia

Technical Appendix

Statistical Model Used to Estimate the Annual Probability of Enterovirus 71 Infection among Children 2–15 Years of Age, Cambodia

For child *i* born in year y_B, the probability of a negative test result on year y_S can be written as:

$$P_i(-|y_S, y_B, \lambda_t) = \exp\left(-\sum_{t=y_B}^{y_S} \lambda_t\right)$$

Where λ_t represents the force of infection at year *t*. Similarly, the probability of being found seropositive on year y_S is given by:

$$P_i(+|y_S, y_B, \lambda_t) = 1 - P_i(-|y_S, y_B, \lambda_t) = 1 - \exp\left(-\sum_{t=y_B}^{y_S} \lambda_t\right).$$

The contribution to the likelihood of case i is therefore $P_i = P_i(+|y_S, y_B, \lambda_t)$, while the contribution of non-case *j* is $P_j = P_j(-|y_S, y_B, \lambda_t)$. The log-likelihood is therefore:

$$L = \sum_k \ln P_k \ .$$

We used a uniform prior for each λ_t and estimated the parameters using a Metropolis-Hastings algorithm in a Markov Chain Monte Carlo (MCMC) framework. (ref: Gilks, W. R., Richardson, S. & Spiegelhalter, D. J. 1996, *Markov Chain Monte Carlo in practice*. London, UK: Chapman and Hall). Average values and 95% credible intervals were obtained from the

posterior distribution for each parameter. The convergence of the MCMC was assessed by inspection of the parameters’ trace plots and acceptance rate plots.

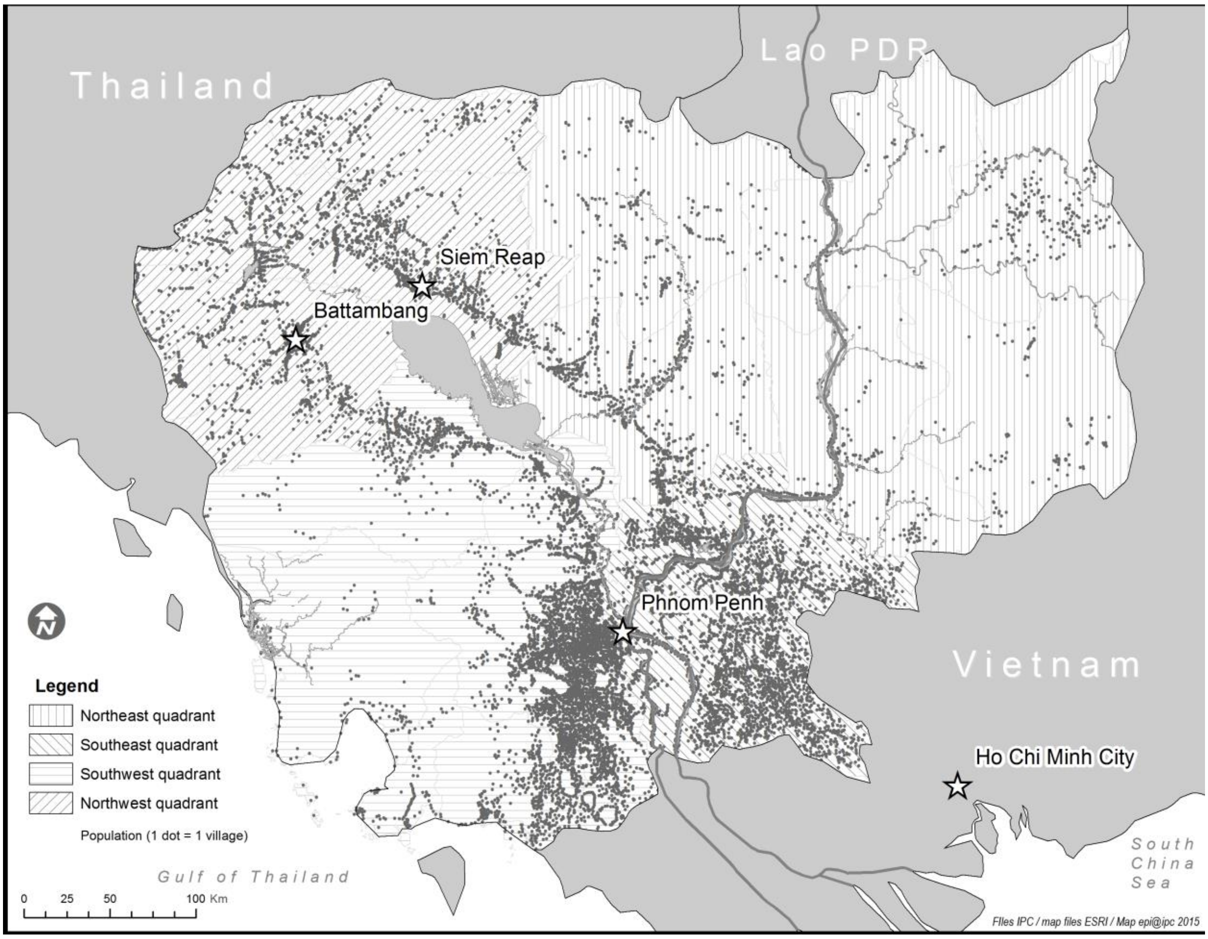

Technical Appendix Figure 1. Distribution of Cambodian provinces into quadrants and underlying population density, mapping villages as proxy (ArcGIS 10, Esri Co., Redlands, CA, USA).

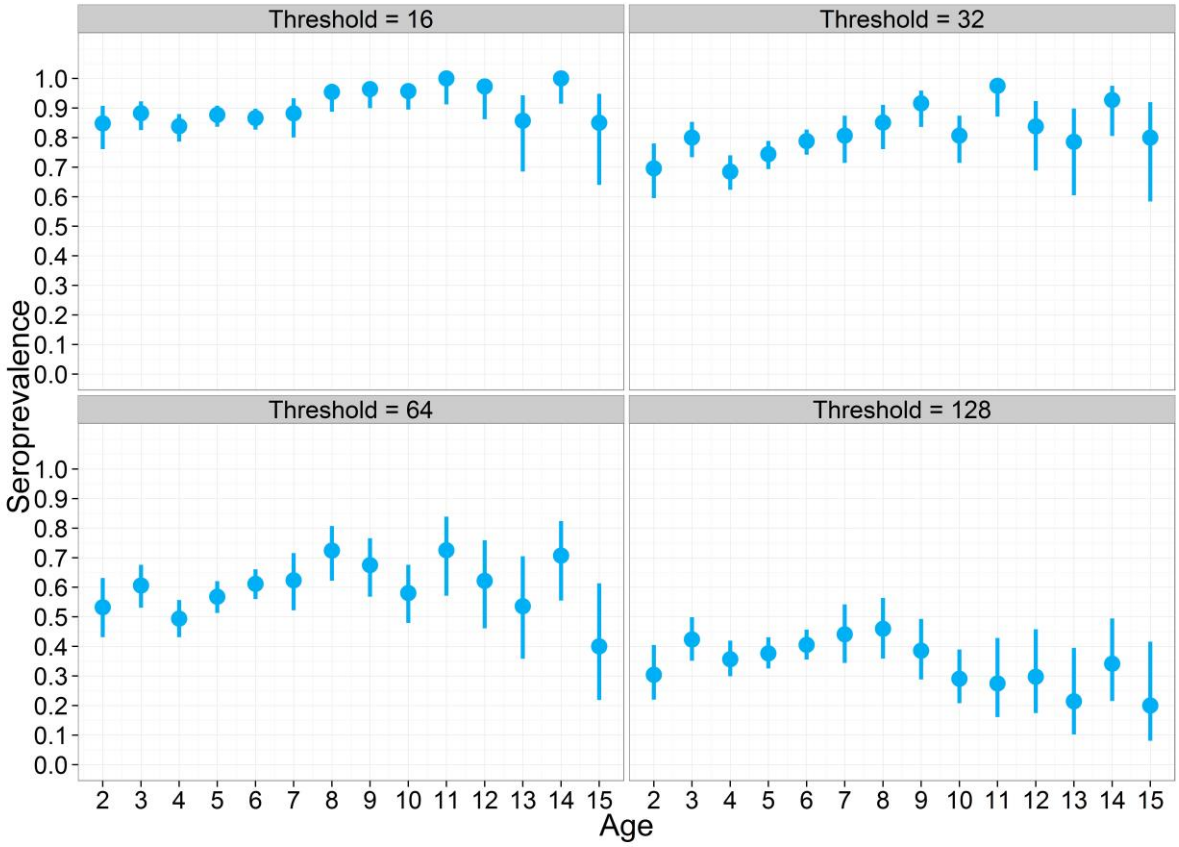

Technical Appendix Figure 2. Age associated seroprevalence among children 2–15 years of age, Cambodia, calculated by using different microneutralization cutoff titers.

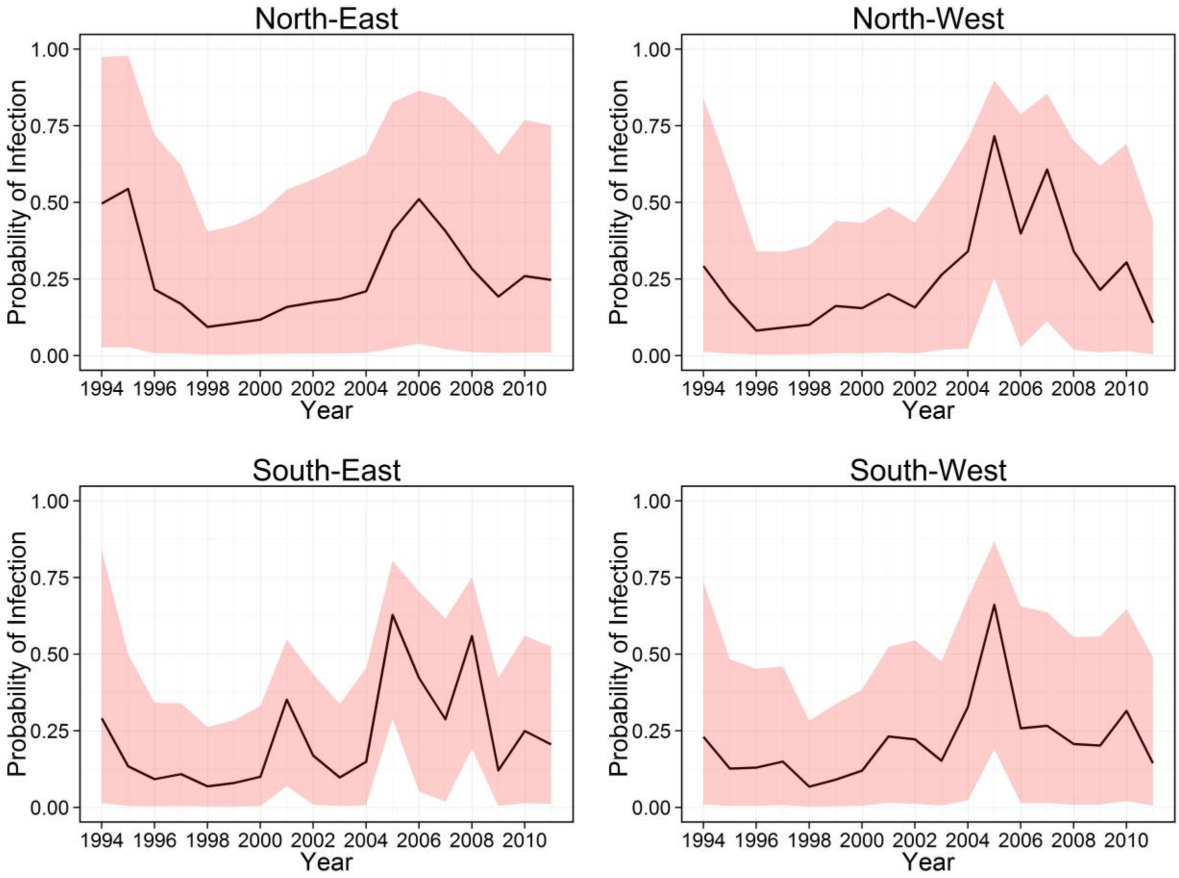

Technical Appendix Figure 3. Annual probability of enterovirus 71 infection among children 2–15 years of age, Cambodia, by geographic quadrant, 1994–2011.
